# A novel, multi-component contingency management intervention in the context of a syndemic of drug-related harms in Glasgow, Scotland: First year of the ‘WAND’ initiative

**DOI:** 10.1016/j.abrep.2024.100580

**Published:** 2024-12-31

**Authors:** S. Smith, K.M.A. Trayner, J. Campbell, A. McAuley, J. Craik, C. Hunter, S. Priyadarshi, S.J. Hutchinson

**Affiliations:** a School of Health and Life Sciences, Glasgow Caledonian University, Glasgow, UK; b Public Health Scotland, Glasgow, UK; c Glasgow Alcohol and Drug Recovery Services, NHS Greater Glasgow and Clyde, Glasgow, UK; d Public Health, NHS Greater Glasgow and Clyde, Glasgow, UK

**Keywords:** Contingency management, Drug-related harms, Harm reduction, People who inject drugs, Public Health

## Abstract

**Background:**

To address high levels of drug-related harms among people who inject drugs (PWID) in Glasgow, a novel contingency management intervention was developed to engage high-risk PWID with four harm reduction measures (known as the WAND initiative: **W**ound care, **A**ssessment of injecting, **N**aloxone, and **D**ried blood-spot test). Our aims were to assess if WAND engaged and re-engaged high-risk PWID.

**Methods:**

Baseline data of WAND participants (n = 831) from 1st Sept-2020 to 30th Aug-2021 were analysed. For those who took part in the first six months, extent of re-engagement was determined and, among those re-engaged, intervention coverage was compared between baseline and final engagement.

**Results:**

Among those who first engaged in WAND from Sept 2020-Feb 2021 (n = 546), 40 % re-engaged by Aug 2021. Compared to those who engaged once (n = 321), those who re-engaged (n = 215) within one year had higher baseline prevalence of injecting risk behaviours such as injecting daily (56 % vs 64 %, p = 0.032), injecting away from home (42 % vs 52 %, p = 0.0179), injecting cocaine (56 % vs 75 %, p < 0.001), having an SSTI (40 % vs 60 %, p < 0.001), and been prescribed naloxone (57 % vs 72 %, p < 0.001). For those who re-engaged six months to one year after initial engagement (n = 107), there was a significant increase in having a BBV test in the last six months (61 % to 81 %, p = 0.003) and carrying naloxone (22 % to 32 %, p = 0.011).

**Conclusion:**

WAND was able to engage and re-engage large numbers of high-risk PWID with multiple harm reduction initiatives. These findings suggest contingency management could be a useful tool for harm reduction services.

## Highlights

• The WAND initiative engaged high-risk PWID in BBV testing and other harm reduction interventions.
• Contingency management can effectively engage high-risk individuals in harm reduction services.
• Incentivisation may be particularly warranted in outbreaks and efforts to eliminate HIV and HCV.

## 1 Introduction

‘Syndemics’ are a conceptual framework for understanding multiple diseases or health conditions that arise in populations and are reinforced by social, economic, environmental, and political population factors (Singer et al., 2017, Singer and Clair, 2003, Tsai et al., 2017). Injecting drug use contributes to a significant burden of disease internationally and people who inject drugs (PWID) experience high rates of concurrent harms, including HIV, Hepatitis C, and overdose (Degenhardt et al., 2023). The prevalence and risk of injecting-related harm is influenced by the context in which they occur, including high rates of social deprivation, the criminalisation of drug use, and the provision of health and social services (Bonn et al., 2020, Degenhardt et al., 2023, Singer et al., 2017, Tsai et al., 2017).

Despite the provision of harm reduction services fundamental for the prevention of opioid related mortality and injecting-related infections, including needle and syringe provision, opiate agonist therapy and take-home naloxone, Scotland is experiencing a syndemic of drug-related harms (McAuley et al., 2023, Public Health Scotland, 2022, Public Health Scotland, 2023). This includes an ongoing drug-related deaths epidemic with rates amongst the highest in Europe as well as globally (European Monitoring Centre for Drugs and Drug Addiction, 2021, National Records of Scotland, 2024). In Greater Glasgow and Clyde, Scotland’s most populous area incorporating the city of Glasgow, drug-related death rates are among the highest nationally (National Records of Scotland, 2024). Additionally, Glasgow has been at the epicentre of major outbreaks of infectious disease among PWID, including anthrax, wound botulism and HIV (McAuley et al., 2019, Palmateer et al., 2012, Trayner et al., 2018). Key risk factors for the drug-related harms among PWID in Glasgow include homelessness, polydrug use (e.g. opioids alongside benzodiazepines and stimulants), and public injecting (McAuley et al., 2019, Trayner et al., 2022). PWID in Glasgow experience higher rates of public injecting than the rest of Scotland, putting them at higher risk for blood-borne virus (BBV), overdose, and injection related complications (Arum et al., 2021, Trayner et al., 2020).

In September 2020, to improve engagement with services and respond to high levels of HIV, drug-related mortality and injection site infections and complications, the regional health authority in Greater Glasgow and Clyde (GGC) designed and implemented a new contingency management intervention in Glasgow City (Scottish Drugs Deaths Taskforce, 2022). The ‘WAND’ initiative offers four harm reduction interventions: Wound care; Assessment of injecting risk; Naloxone; Dry blood spot testing. If an individual completes all four WAND components, they become eligible to receive a £20 cash voucher.

As the WAND initiative involves compensation for engaging in harm reduction services, it is considered a contingency management (CM) intervention (i.e., an intervention that involves financial incentives). Following the introduction of WAND in NHS GGC, Scottish Government recommended expanding CM initiatives to other health boards (Scottish Government, 2021); a CM initiative called ‘Cocoon’ has also been piloted in Dundee, Scotland (Byrne et al., 2023). Systematic reviews and *meta*-analyses on the effectiveness of contingency management for PWID have reported significant reductions in, and increased abstinence from, illicit drug use, improved adherence to medication assisted treatment (MAT), and better attendance at harm reduction services (Bentzley et al., 2021, Bolívar et al., 2021, De Crescenzo et al., 2018, Herrmann et al., 2017). Most CM initiatives focus on reinforcing one desired behaviour (i.e., abstinence, adherence or attendance), whereas the WAND initiative uniquely necessitates engagement with four harm reduction interventions, providing compensation upon completion of all four elements.

The principal objective of WAND is to engage a highly marginalised group, at risk of severe drug-related harms, in regular harm reduction interventions to improve both individual health and overall public health within the city. WAND was developed to engage PWID at high risk and not regularly engaged with routine services in order to address the high levels of drug related harm in Glasgow (McAuley et al., 2023, Public Health Scotland, 2022, Public Health Scotland, 2023). To assess whether WAND was able to engage high risk individuals, we compared characteristics and injecting-related harms of those engaged with the intervention to PWID surveyed in Glasgow through an established bio-behavioural surveillance initiative of this population (known as the Needle Exchange Surveillance Initiative (NESI) (Public Health Scotland et al, 2022)).

There is limited research assessing the use of CM to engage PWID in multiple low threshold harm reduction interventions. Utilising available service data, we aimed to assess if WAND was able to engage and re-engage a high-risk group of PWID in Glasgow.

## 2 Methods

### 2.1 Intervention

The WAND initiative is provided by three fixed site non-governmental organisations and one outreach mobile needle and syringe provision (NSP) van in Glasgow City Centre. All staff are required to complete the Advanced Harm Reduction Workers Training Programme (AHRWTP), a comprehensive training programme to ensure staff delivering the WAND initiative are competent in conducting the range of involved interventions. PWID are eligible to participate in WAND if they have recently accessed injecting equipment or have evidence of current or recent injecting. The following are the four harm reduction interventions offered through WAND for people who currently inject drugs:

• **W**ound care: Staff examine a participant’s injecting site(s) and if a complication is identified the patient is offered wound first aid;
• **A**ssessment of injecting risk (AIR): Staff ask participants a series of questions related to their injecting practices and create a care plan to reduce harm based on their responses;
• **N**aloxone: Staff ask if the participants are in possession of an unopened naloxone kit. If not, the participant is provided with naloxone and encouraged to carry it with them;
• **D**ry blood spot (DBS) test: Participants provide a dried blood spot which is tested for HIV and HCV.

After completing all four interventions, each participant receives a £20 cash voucher. After initial engagement with WAND, participants were encouraged to return every three months, which is the minimum period they must wait before re-engaging and earning further cash compensation. Their participation is tracked by a unique identifier, which is allocated to all individuals accessing services for injecting equipment and other harm reduction interventions in Glasgow.

### 2.2 Service level data

Data gathered by NHS Greater Glasgow and Clyde for all people who engaged in the WAND initiative during its first year (from September 2020 to August 2021), as well as one year follow-up data through to February 2022 for those who engaged with WAND in the first six months (from September 2020 to February 2021), were included.

The people who engaged in WAND completed an assessment of injecting risk (AIR) as one of the four interventions. The administrative data collected from the AIR tool and recorded on the neo360 database, a commercial database used by NSP sites across mainland Scotland to record NSP attendance and injecting equipment distribution (Neo360., 2023, Public Health Scotland, 2023), provided the basis for this descriptive retrospective cohort analysis. Before commencing the study, we assessed our protocol against the 'defining research' tool (Health Research Authority, 2022) and concluded that we did not require NHS ethics approval as this investigation fell under the service evaluation category as it involved analysis of existing retrospective service level data, which were anonymised prior to analysis.

The AIR tool includes questions on participants’ injecting practices (i.e., frequency of injecting, types of drugs used, frequency of sharing injecting equipment). A subset of key data on frequency of injecting (ie, once or more daily); ever injected away from home; ever shared injecting equipment; whether had a skin or soft tissue infection (SSTI) in the last six months, types of drugs used (ie, cocaine injected in the last six months); naloxone carriage (ie, prescribed naloxone in the last year); and awareness of BBV status from the AIR tool were compared to equivalent data collected from the Needle Exchange Surveillance Initiative (NESI) (Glasgow City recruitment sites only) (Public Health Scotland et al., 2022).

Data from the AIR tool provided the baseline characteristics of participants in the first year of WAND. Using the AIR data, we also calculated the proportion of those who initially engaged with WAND in the first six months who subsequently re-engaged within one year. Blood-borne virus (BBV) test results recorded in neo360 for those who initially engaged in the first year of WAND were used to calculate the proportion tested for, and tested positive with, HIV and HCV. Some BBV test results were recorded on NHS systems rather than neo360 and as there was no unique identifier held on both systems to facilitate data linkage, and thus they were excluded from the analysis. In addition, individuals known to be infected and diagnosed were not re-tested.

### 2.3 Statistical analysis

We compared baseline characteristics for those who only engaged with WAND once versus those who re-engaged with WAND on more than one occasion. We used the two-sample z test for proportion to test against the null hypothesis for no difference in population proportions estimated between those who engaged once versus those who re-engaged with WAND on more than one occasion and to determine any significant differences between the two groups. The rationale for this, was to see if WAND was able to continue to engage those with high-risk behaviours and determine whether the two populations differed significantly on specific characteristics.

In order to examine changes in important measures for those engaged in WAND, we also compared key indicators (e.g., testing for HIV or HCV in the last six months and carriage of naloxone on the day of assessment) at baseline and final follow up for individuals who initially engaged with WAND during the first six months and had at least one follow-up engagement six months to one year after their initial engagement. As this involved two dependent samples (i.e., the same individuals responding at two different points in time) we used McNemar's chi-square test for comparing dichotomous variables in two dependent samples to determine any significant differences between these indicators at baseline and final follow-up.

All data were analysed in Stata version 13.1 (StataCorp, 2013) and Microsoft Excel 365 (Microsoft Corporation, 2021).

## 3 Results

### 3.1 Engagement with WAND and baseline characteristics

A total of 831 individuals engaged with WAND in the first year, from September 2020 to August 2021. Most (55 %, n = 459) engaged for the first time in the first three months from September to November 2020, followed by 87 (10 %) in December 2020-February 2021, 176 (22 %) in March-May 2021, and 109 (13 %) in June-August 2021. Overall, the majority were male (78 %; n = 646), which increased to 89 % for those attending from December 2020 to February 2021. Individuals were mainly aged 30–39 (37 %, n = 307) or 40–49 years old (38 %, n = 318), though from March to May 2021 almost half were 40–49 (47 %) (Table 1).

**Table 1:** Baseline characteristics at first engagement with the WAND initiative from September 2020 to August 2021, stratified by 3-month intervals: Sept 2020-Feb 2021, March–May 2021, and June–Aug 2021.

	Sept-Nov 2020 N(%N)	Dec 2020 – Feb 2021 N(%N)	March – May 2021 N(%N)	June – Aug 2021 N(%N)	Total N(%N)
Total n(%N)	459 (100)	87 (100)	176 (100)	109 (100)	831 (100)

Age					
<20–29	52 (11)	10 (12)	16 (9)	16 (15)	94 (11)
30-39	188 (41)	33 (38)	46 (26)	40 (37)	307 (37)
40-49	163 (36)	32 (37)	82 (47)	41 (38)	318 (38)
50+	56 (12)	12 (14)	32 (18)	12 (11)	112 (14)

Gender					
Male	369 (78)	77 (89)	130 (74)	79 (72)	646 (78)
Female	99 (22)	10 (11)	46 (26)	30 (28)	185 (22)

Frequency of injecting at time of interview					
Daily or more	277 (60)	48 (55)	72 (41)	66 (61)	463 (56)
Less than Daily	182 (40)	39 (44)	104 (59)	43 (39)	368 (44)

Ever injected away from home^a^					
Yes	210 (46)	42 (48)	62 (35)	51 (47)	365 (44)
No	249 (54)	45 (52)	114 (65)	58 (53)	466 (56)

Any skin and soft tissue infection/complication over the past 6 months					
Yes	214 (47)	50 (57)	106 (60)	69 (63)	439 (53)
No	245 (53)	37 (43)	70 (40)	40 (37)	392 (47)

Overdosed in the last year					
Yes	150 (33)	33 (38)	53 (30)	43 (40)	279 (34)
No	309 (67)	54 (62)	123 (70)	66 (61)	552 (66)
Ever shared or re-used own needles, syringes or equipment					

*Shared needles/syringes*					
Yes	90 (20)	14 (16)	33 (19)	13 (12)	150 (18)
No	369 (80)	73 (84)	143 (81)	96 (88)	681 (82)

*Shared spoons, water, or filter*					
Yes	145 (32)	30 (35)	79 (45)	56 (51)	310 (37)
No	314 (68)	57 (66)	97 (55)	53 (49)	521 (63)

*Reused own needle/syringe*					
Yes	226 (49)	55 (63)	103 (59)	63 (58)	447 (54)
No	233 (51)	32 (37)	73 (42)	46 (42)	384 (46)

Benzodiazepine injected or swallowed over the last 6 months					
Yes	274 (60)	59 (68)	110 (63)	70 (64)	513 (62)
No	185 (40)	28 (32)	66 (38)	39 (36)	318 (38)

Cocaine injected over the last 6 months^b^					
Yes	281 (61)	66 (76)	115 (65)	77 (71)	539 (65)
No	178 (39)	21 (24)	61 (35)	32 (29)	292 (35)

Crack/freebase cocaine smoked over the last 6 months					
Yes	50 (11)	10 (12)	39 (22)	19 (17)	118 (14)
No	409 (89)	77 (88)	137 (78)	90 (83)	713 (86)

Heroin injected over the last 6 months					
Yes	355 (77)	68 (78)	132 (75)	82 (75)	637 (77)
No	104 (23)	19 (22)	44 (25)	27 (25)	194 (23)

Prescribed take home naloxone in last year					
Yes	291 (63)	55 (63)	101 (57)	57 (52)	504 (61)
No	168 (37)	32 (37)	75 (43)	52 (48)	327 (39)

“Aware” of HIV status at time of assessment					
Yes	179 (39)	32 (37)	52 (30)	27 (25)	290 (35)
No	280 (61)	55 (63)	124 (70)	82 (75)	541 (65)

“Aware” of HCV status at time of assessment					
Yes	193 (42)	42 (48)	53 (30)	31 (28)	319 (38)
No	266 (58)	45 (52)	123 (70)	78 (72)	512 (62)

Note.

a Ever injected away from home includes friend home, shelter/hostel, prison, public toilet, car park, stairwell, outdoor park/alleyway, squat/abandoned house.

b Cocaine injected includes cocaine powder, cocaine freebase/crack, and cocaine + heroin (snowball).

### 3.2 Assessment of injecting risk (AIR)

Over half (56 %) of people engaging in the first year of WAND (n = 463) reported injecting daily or more at the time of their interview. Overall, nearly half (44 %, n = 365) reported ever injecting away from home; 54 % (n = 447) reported ever re-using their own needle/syringe; and 37 % (n = 447) reported ever sharing injecting equipment such as spoons, water, or filter. A high proportion reported heroin injecting (77 %, n = 637), cocaine injecting (65 %, n = 539), and benzodiazepine use (62 %, n = 513) in the last six months. Around half (53 %, n = 439) reported an SSTI in the last six months and a third (34 %, n = 279) reported an overdose in the last year. Just over a third 35 % (n = 290) reported that they were aware of their HIV status and 38 % (n = 319) were aware of their HCV at status at baseline (Table 1).

**Fig. 1:**
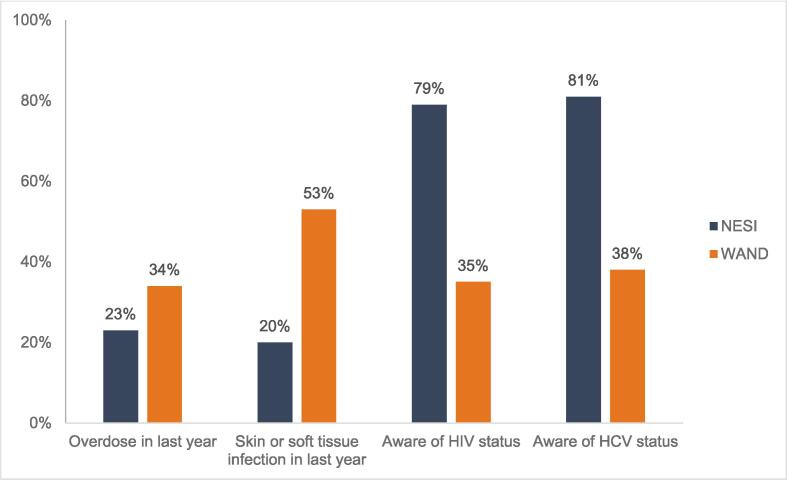
Comparison of equivalent baseline characteristics from WAND (n = 831) and the Needle Exchange Surveillance Initiative, NESI (n = 715), Glasgow City recruitment sites only.

When compared to participants who took part in NESI (Glasgow City recruitment sites only), WAND participants reported lower awareness of their HIV and HCV status and were more likely to report an overdose (23 % vs 34 %, respectively) and an SSTI or complication in the last year (20 % vs 53 %, respectively) (Fig. 1/Supp. Table 1).

### 3.3 BBV testing

Among the total who took part in the first year of WAND (n = 831), baseline BBV test results were captured for 632 (76 %) individuals. Of those with baseline BBV test results, 83 % (n = 527) had an HIV test result, and of those, 4 % (n = 21) were HIV positive; of these 21 cases, the majority were previously diagnosed and ∼ 10 % (<5) were newly diagnosed. For HCV, 82 % (n = 517) had baseline BBV test results had an HCV test result, and of those, 50 % (n = 258) tested antibody negative (RNA negative or RNA not tested) or RNA negative (antibody not tested), 28 % (n = 145) tested antibody positive (RNA negative or RNA not tested), and 22 % (n = 114) tested RNA positive (antibody positive or antibody not tested). For those who tested positive for HCV, we were unable to determine if they were newly diagnosed cases.

### 3.4 Baseline characteristics associated with re-engagement

**Fig. 2:**
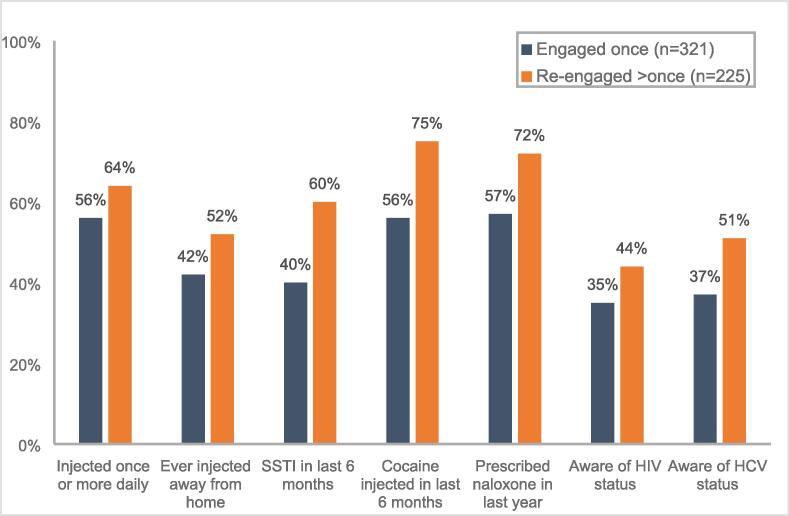
Comparison of baseline characteristics for those who engaged with WAND once (n = 321) versus those who engaged with WAND one more than one occasion (n = 225), of those who initially engaged with WAND from September 2020 − February 2021 (n = 546). *Note.* Ever injected away from home includes friend home, shelter/hostel, prison, public toilet, car park, stairwell, outdoor park/alleyway, squat/abandoned house.

Of the 546 who engaged in the first six months of WAND (September 2020 to February 2021), 41 % (n = 225) re-engaged within one year; 52 % (n = 118) re-engaged within six months and 48 % (n = 107) in the subsequent six months. We compared baseline characteristics for those who only engaged with WAND on one occasion (n = 321) to those who re-engaged with WAND on more occasions (n = 225) within one year. Compared to those who only engaged with WAND once, those who re-engaged on more than one occasion had a higher prevalence of injecting risk behaviours such as injecting daily (56 % vs 64 %, p = 0.032), injecting away from home (42 % vs 52 %, p = 0.0179), and injecting cocaine in the last six months (56 % vs 75 %, p < 0.001). Those who re-engaged on more than one occasion were also more likely to have had an SSTI in the last six months (40 % vs 60 %, p < 0.001) and be prescribed naloxone in the last year (57 % vs 72 %, p < 0.001). Additionally, baseline awareness of HIV (35 % vs 44 %, p = 0.025) and HCV (37 % vs 51 %, p = 0.001) status was higher among those who re-engaged (Fig. 2/ Supp. Table 2).

### 3.5 Comparison of key indicators at baseline and follow-up

Lastly, we assessed if there was any change in key indicators between baseline and follow-up for those who initially engaged with WAND services in the first six months and had at least one follow-up engagement six months to one year after their initial engagement (n = 107); for the latter, the last engagement in this period was used. Reporting of SSTI over the past six months was similar at baseline (64 %) and follow-up (61 %) (p = 0.654). While 55 % indicated they had been tested for HIV and 58 % for HCV within the last six months at baseline, which increased to 75 % and 76 %, respectively, at final follow-up (HIV: p = 0.002, HCV: p = 0.004). The proportion who reported carrying naloxone on the day of assessment increased from 16 % at baseline to 32 % at final follow-up (p = 0.011) (Table 2).

**Table 2:** Key indicators at baseline and final follow-up of those who first engaged with WAND services from September 2020 to February 2021 and had at least one follow-up engagement six months to one year after their initial engagement (n = 107).

	Recruited September 2020–February 2021		
	Baseline	Final follow up	P Value^a^
SSTI in the past 6 months	68 (64)	65 (61)	0.654
HIV test in last 6 months	59 (55)	80 (75)	0.002
HCV test in last 6 months	62 (58)	81 (76)	0.004
Naloxone carriage on day of assessment	17 (16)	34 (32)	0.011
Total n(%N)	107 (100)	107 (100)	−

Note.

a McNemar's chi-square test for paired data.

## 4 Discussion

The WAND initiative, a CM intervention, was implemented in response to a syndemic of drug-related harm in Glasgow, aiming to engage PWID at high risk and not regularly engaged with routine services. We found that WAND was able to engage a high-risk population of PWID in Glasgow City illustrated by the high proportions of those engaged unaware of their BBV status at baseline, and high prevalence of key risk behaviours. Whilst re-engagement in WAND every three months was encouraged, we found that less than half (41 %) of WAND participants re-engaged within one year of first engagement.

In response to the high rates of drug-related harm in Scotland, the Scottish Government has published a National Mission in addition to Medication Assisted Treatment (MAT) standards, both of which include a specific focus on improving access and retention in harm reduction services for PWID (Scottish Government, 2022a, Scottish Government, 2022b). The Scottish Drug Death Taskforce also recommended WAND be expanded into primary care and drug treatment services throughout Scotland (Scottish Drugs Deaths Taskforce, 2022). Similar initiatives to WAND (that utilise contingency management) have been implemented in other parts of Scotland, including a nurse-led holistic care pathway in Dundee (‘Cocoon’) that offers PWID a £20 voucher to engage in a health assessment alongside BBV testing every six months (Byrne et al., 2023, NHS Tayside, 2022), and are being considered for implementation more widely.

The majority of WAND participants reported injecting heroin in the last six months (77 %) and a high proportion reporting injecting cocaine (65 %), a key driver of the HIV outbreak in Glasgow (McAuley et al., 2019, Trayner et al., 2020), as well as benzodiazepine use (62 %), a contributing factor in the increase in drug-related deaths (McAuley et al., 2022, National Records of Scotland, 2024). Baseline findings from the Cocoon care pathway suggest it was similarly able to engage a high-risk population of PWID, with 36 % sharing injecting equipment, 83 % injecting daily, and 36 % reporting a recent non-fatal overdose (Byrne et al., 2023, NHS Tayside, 2022). In addition, WAND participants reported a higher baseline prevalence of a recent non-fatal overdose (34 %) and SSTIs (53 %), when compared to those surveyed at pharmacies and other NSP services (through NESI) suggesting the intervention is engaging those at highest risk of drug-related harms in Glasgow. Furthermore, these baseline prevalence estimates were higher than global prevalence estimates of people who use drugs for recent non-fatal overdose (20.5 %) and an SSTI in the last 6–12 months (7–35 %) (Colledge et al., 2019, Larney et al., 2017).

Our findings show that less than half of WAND participants (41 %) re-engaged within one year. Data on re-engagement from the Cocoon care pathway are not yet publicly available, but preliminary findings suggest that the use of CM may have been beneficial for encouraging re-engagement. Research on CM to encourage hepatitis B vaccine uptake among PWID found the use of incentives both improved vaccine uptake as well as timely appointment attendance, highlighting CM to be an effective method for re-engaging PWID (Topp et al., 2013, Weaver et al., 2014). Our results also indicate that those who re-engaged appeared to be a higher risk population as they were more likely to report injecting daily, injecting away from home, and injecting cocaine (a key driver of HIV in Glasgow). They were also more likely to report having had an SSTI in the last six months and being prescribed naloxone in the last year, indicating that WAND facilitated ongoing engagement with high-risk PWID. Given the high levels of cocaine injecting within those engaged with WAND, expanding CM in Glasgow could be considered to address the high levels of cocaine use, as a recent global systematic review found that CM is the only intervention associated with a reduction in stimulant use (Farrell et al., 2019).

WAND was implemented during the COVID-19 pandemic, which may have affected re-engagement. During the first wave of COVID-19 in Scotland, both NSP provision and BBV testing reduced significantly, whilst OAT prescribing remained relatively stable (Trayner et al., 2022). As such, the pandemic may have increased the risk of drug-related harm including BBV transmission, due to the widespread disruption to harm reduction and BBV testing services (Trayner et al., 2022). Thus, implementation of the WAND initiative at that time may have helped mitigate some of the increased risks in harm by encouraging people to attend and engage with harm reduction services (Williams et al., 2022).

Scottish Government (SG) aims to eliminate hepatitis C virus by March 2025 and HIV transmission by 2030 (Scottish Government., 2019b, Scottish Government, 2022a). Initiatives like WAND aim to help support this goal by more effectively reaching and regularly testing PWID populations at high risk of (re-)infection (Robinson et al., 2023, Scottish Government, 2022a, Scottish Government., 2019b). This is in line with updated WHO guidance, which supports simplifying care pathways with delivery of HCV testing and treatment at community-based facilities (World Health Organization, 2022). Although WAND was not able to determine if those who tested positive for HCV were newly diagnosed cases due to issues with data linkage, it was able to successfully identify new cases of HIV among a marginalised injecting population. Future research will assess the role of WAND in detecting HCV (re-)infection and linkage to treatment/care.

In the context of an HIV outbreak in Glasgow along with high rates of HCV among PWID, engagement with testing services is critical for controlling the HIV outbreak as well as successfully treating those infected with HCV (Pourmarzi et al., 2020, Trayner et al., 2021). As the COVID-19 pandemic response greatly impacted access to BBV testing throughout Scotland, it is vitally important for initiatives to improve access to testing for those with ongoing risk for HIV and HCV (Scottish Government, 2021, Trayner et al., 2022). While there is limited research on the use of CM to encourage HIV testing among PWID, the ARISTOTLE combination intervention implemented in Athens, Greece in response to an outbreak of HIV among PWID highlights the potential role CM could play in encouraging HIV testing in high-risk populations. ARISTOTLE, which used a combination of incentives and respondent driven sampling to recruit PWID, was able to recruit high levels of PWID and saw a significant decrease in the proportion of undiagnosed infection (Hatzakis et al., 2015, Sypsa et al., 2017). Those engaged with WAND in the first six months reported higher rates of having had a test for HIV and HCV at final follow-up compared to baseline, indicating WAND may be an effective tool for increasing BBV testing among this population. Additionally, a recent a systematic review found mixed evidence that incentive-based interventions can improve HCV testing and treatment uptake, reflecting the need for more research involving comparative study designs to assess the effectiveness of CM approaches to engage PWID in harm reduction interventions (Shen et al., 2024).

Previous CM trials have targeted abstinence from drugs as well as treatment adherence (e.g., appointment attendance, retention and hepatitis B vaccination) (Bentzley et al., 2021; Bolívar complet al., 2021; De Crescenzo et al., 2018). CM is typically used as a psychosocial intervention alongside other treatment interventions such as methadone maintenance to improve treatment outcomes and is recommended by National Institute for Health and Care Excellence (NICE) to promote abstinence from illicit drugs and improve engagement with services (National Institute for Health and Clinical Excellence NICE, 2007). However, there is limited research on the use of CM to engage PWID with multiple low threshold harm reduction services, without necessitating abstinence or adherence to drug treatment, making WAND a novel intervention. The individual elements of WAND were previously available as separate components of harm reduction and treatment services, with WAND utilising CM to facilitate engagement with those existing services. WAND is also unique in that it offers a cash voucher that can be spent without restriction, whereas other initiatives engaging PWID typically offer shopping vouchers that can only be redeemed at grocery stores (Byrne et al., 2023, NHS Tayside, 2022; Public Health Scotland et al., 2022). Research has suggested that the provision of cash, rather than store vouchers, is more effective in engaging PWID (Topp et al., 2013). The combination of high levels of drug-related harm and wide coverage of harm reduction services makes Scotland an ideal location for an intervention like WAND. However, introducing a similar CM intervention in other parts of the world may depend on availability of such services and resourcing of the incentivised approach.

A key component of Scotland’s National Mission to reduce drug-related mortality is improving take-home naloxone provision (Public Health Scotland, 2022). To the best of our knowledge, there is limited research assessing the use of CM to increase naloxone coverage and carriage. Findings from WAND showed a higher proportion carrying naloxone at final follow-up for those who re-engaged. This is an important outcome from WAND, as 34 % reported experiencing an overdose in the last year and evidence shows take-home naloxone to be an effective way to reduce opioid-related mortality (Giglio et al., 2015, McDonald and Strang, 2016). Similarly, the Cocoon care pathway found that 72 % of those who engaged accepted take-home naloxone from practitioners, indicating that CM may be an acceptable way of both increasing naloxone uptake and carriage (Byrne et al., 2023). Future research is needed to further assess the effect of CM on naloxone coverage and carriage.

### 4.1 Limitations

Our findings from the first year of the WAND initiative have shown promising results, however, this evaluation had a number of limitations. Firstly, the AIR tool used in WAND to collect data on participants’ injecting practices involved several different recall periods throughout the assessment (i.e., in the last six months/in the last year/ever). As participants were encouraged to attend WAND services every three months, this resulted in follow-up data falling within those recall periods making it difficult to track any changes over time. Secondly, the AIR tool relies heavily on self-report data which may have been affected by recall bias. There were also significant challenges linking the WAND BBV testing data, which involved anonymous testing in third sector services, to routine laboratory data to determine the extent of new diagnoses. BBV tests conducted at NHS sites were not available for analysis, potentially skewing re-testing figures and contributing to high levels of missing data, as a test record at baseline or follow-up may be missing. However, improvements to data linkage have since been made to allow for better follow up and management for those diagnosed with BBVs through WAND. Future work, including qualitative research, is warranted to fully assess the acceptability and barriers to implementing an incentivised approach to engaging PWID in harm reduction services across Scotland.

Another limitation is that it was not feasible to assess whether or not the requirement to complete four harm reduction interventions before receiving compensation affected engagement, however, there is some evidence that providing financial incentives quickly is important in contingency management as it helps prevent delay discounting, which is the tendency among PWID to devalue positive reinforcement that is not provided immediately (Businelle et al., 2010, McPherson et al., 2018).

## 5 Conclusion

There is limited research internationally that aims to assess the use of contingency management to engage with multiple low threshold harm reduction services. We have assessed the use of a novel contingency management intervention, WAND, to engage a highly marginalised group of people at risk of severe drug-related harms in existing harm reduction and treatment services. Our findings show that in its first year of implementation, the WAND initiative was successful in engaging a large number of PWID at risk for drug related harms during the pandemic. Additionally, a substantial minority re-engaged with WAND services, within one year of their first engagement. Further research is required to fully evaluate WAND, however, these preliminary findings suggest that contingency management could be a useful tool to engage PWID with harm reduction services, both in Scotland and internationally.

## Ethical approval

The authors declare that the work reported herein did not require ethics approval as it involved secondary analysis of routine administrative data.

## Funding sources

This research was supported by Public Health Scotland. The funders did not influence the design of the study, the collection, analysis or interpretation of data, the writing of the report, or the decision to submit for publication.

## CRediT authorship contribution statement

**S. Smith:** Writing – review & editing, Writing – original draft, Visualization, Formal analysis, Data curation. **K.M.A. Trayner:** Writing – review & editing, Formal analysis. **J. Campbell:** Writing – review & editing, Resources, Conceptualization. **A. McAuley:** Writing – review & editing, Supervision, Conceptualization. **J. Craik:** Writing – review & editing, Resources, Conceptualization. **C. Hunter:** Writing – review & editing. **S. Priyadarshi:** Writing – review & editing. **S.J. Hutchinson:** Writing – review & editing, Supervision, Conceptualization.

## Declaration of competing interest

The authors declare that they have no known competing financial interests or personal relationships that could have appeared to influence the work reported in this paper.

## Data Availability

The authors do not have permission to share data.
