# Supplementary material for: A novel, multi-component contingency management intervention in the context of a syndemic of drug-related harms in Glasgow, Scotland: First year of the ‘WAND’ initiative
Source: Addict Behav Rep. 2024 Dec 31;21:100580. doi: 10.1016/j.abrep.2024.100580 (PMC11761270; doi:10.1016/j.abrep.2024.100580)
Supplement: Supplementary Data 1 [file mmc1.docx]

**Supplementary Material**

**Supplementary Table 1**

*Summary of indicators from the Needle Exchange Surveillance Initiative (NESI) for comparison to WAND, 2019-20 (pre-pandemic)*

|  | Glasgow City council |
| --- | --- |
| Total n(%N)^a^ | 560 |
| Gender | |
| Male | 425 (76%) |
| Female | 134 (24%) |
| Missing/not recorded | 1 (<1%) |
| Age | |
| <35 | 125 (22%) |
| 35-44 | 271 (49%) |
| 45+ | 163 (29%) |
| Missing/not recorded | 1 (<1%) |
| Injected heroin in last six months | |
| Yes | 484 (86%) |
| No | 76 (14%) |
| Missing/not recorded | 0 |
| Injected cocaine in last six months | |
| Yes | 315 (56%) |
| No | 245 (44%) |
| Missing/not recorded | 0 |
| Benzodiazepine (injected, swallowed or snorted) use in last six months |  |
| Yes | 293 (52%) |
| No | 267 (48%) |
| Missing/not recorded | 0 |
| Shared needles/syringes in last six months |  |
| Yes | 71 (12%) |
| No | 480 (86%) |
| Missing/not recorded | 9 (2%) |
| Re-used needles/syringes in last six months | |
| Yes | 252 (45%) |
| No | 293 (52%) |
| Missing/not recorded | 15 (3%) |
| Injecting frequency | |
| Daily or more | 328 (59%) |
| Less than daily | 230 (41%) |
| Missing/not recorded | 2 (<1%) |
| Overdosed in the last year | |
| Yes | 129 (23%) |
| No | 419 (75%) |
| Missing/not recorded | 12 (2%) |
| Skin and soft tissue infection in the last year |  |
| Yes | 112 (20%) |
| No | 440 (79%) |
| Missing/not recorded | 8 (1%) |
| Aware of HIV status | |
| Yes | 442 (79%) |
| No | 110 (20%) |
| Missing/not recorded | 8 (1%) |
| Aware of HCV status | |
| Yes | 455 (81%) |
| No | 100 (18%) |
| Missing/not recorded | 5 (1%) |
| *Note*. ^a^Only includes PWID who reported injecting in the last six months. |  |

**Supplementary Table 2**

*Comparison of baseline characteristics for those who engaged with WAND once (n = 321) versus those who engaged with WAND one more than one occasion (n = 225), of those who initially engaged with WAND from September 2020 - February 2021 (n = 546)*

|  | Recruited September 2020 - February 2021 | | |
| --- | --- | --- | --- |
|  | Engaged once | Engaged on more than one occasion | P value^a^ |
| Total n(%N) | 321 (100) | 225  (100) | - |
| Injected once or more daily | 179 (56) | 146 (64) | 0.032 |
| Ever injected away from home^b^ | 134 (42) | 117 (52) | 0.018 |
| SSTI in last 6 months | 128 (40) | 136 (60) | < 0.001 |
| Cocaine injected in last 6 months | 178 (56) | 168 (75) | < 0.001 |
| Prescribed naloxone in last year | 184 (57) | 163 (72) | < 0.001 |
| Aware of HIV status | 111 (35) | 99 (44) | 0.025 |
| Aware of HCV status | 119 (37) | 115 (51) | < 0.001 |
| *Note.* ^a^Two sample z test of proportions against the null hypothesis for no difference in population proportions estimated from two samples. ^b^Injecting away from home includes friend home, shelter/hostel, prison, public toilet, car park, stairwell, outdoor park/alleyway, squat/abandoned house. | | | |
